# Gender Discrepancies in SARS-CoV-2 Pandemic Related Beliefs, Attitudes, and Practices

**DOI:** 10.3389/fpubh.2021.711460

**Published:** 2021-09-27

**Authors:** Bethann Mangel Pflugeisen, Jin Mou

**Affiliations:** Institute for Research and Innovation, MultiCare Health System, Tacoma, WA, United States

**Keywords:** COVID-19, gender, health equity, social determinants of health (MeSH), coronavirus pandemic (COVID-19 pandemic), SARS-CoV-2

## Abstract

**Objectives:** International studies suggest that males may be less likely to adhere to SARS-CoV-2 transmission mitigation efforts than females. However, there is a paucity of research in this field in the United States. The primary aim of this study was to explore the relationship of binary gender identity (female/male) with beliefs, attitudes, and pandemic-related practices in the early stages of the pandemic.

**Methods:** This study is based on a cross-sectional, voluntary response survey. Patients who were tested for SARS-CoV-2 between March 5 and June 7, 2020 were invited to participate. All patients were tested within a large community healthcare system that serves patients through eight hospitals and hundreds of clinics across Washington State. Bivariate associations between gender and various demographics were tested using Chi-squared and Student's *t*-tests. We examined associations between gender and pandemic-related beliefs, attitudes, and practices using multivariable logistic regression, accounting for potential confounding factors.

**Results:** Females were more likely than males to agree that they (aOR = 1.51, 95% CI 1.14–2.00) or their families (aOR = 1.75, 95% CI 1.31–2.33) were threatened by SARS-CoV-2, or that their own behavior could impact transmission (aOR = 2.17, 95% CI 1.49–3.15). Similarly, females were more likely to agree that social distancing (aOR = 1.72, 95% CI 1.19–2.46), handwashing (aOR = 3.27, 95% CI 2.06–5.21), and masking (aOR = 1.41, 95% CI 1.02–1.94) were necessary to slow SARS-CoV-2 spread. Females were significantly less likely to visit outside of their social distancing circle (aOR = 0.62, 95% CI 0.47–0.81), but among those who did, practices of social distancing (aOR = 1.41, 95% CI 0.89–2.23), remaining outdoors (aOR = 0.89, 95% CI 0.56–1.40), and masking (aOR = 1.19, 95% CI 0.74–1.93) were comparable to males, while females practiced handwashing more than males (aOR = 2.11, 95% CI 1.33–3.34).

**Conclusions:** Our study suggests that gender disparate beliefs, attitudes, and practices existed in the early stages of the SARS-CoV-2 pandemic. Efforts should be tailored to encourage males to engage with mitigation efforts in ongoing pandemic-related public health campaigns.

## Introduction

During 2020 nearly 20 million confirmed cases of the novel SARS-CoV-2 virus were reported to the United States (US) Centers for Disease Control (CDC) and over 300,000 of these resulted in death from SARS-CoV-2 disease (COVID-19). In January 2021, an average of 3,080 people in the US died every day from COVID-19 (1), far outstripping the 9/11 death toll on a daily basis. This highly contagious virus is spread through person-to-person contact, predominantly through inhaled particles released during an infected person's exhalation or, less so, by touching an infected surface and then touching one's face (2). Much of SARS-CoV-2's success is due to facile viral transmission and a high prevalence of asymptomatic spread (3), making physical distancing of six or more feet; remaining outdoors whenever possible; frequent, thorough handwashing; and masking of the mouth and nose public health hallmarks of the pandemic.

Understanding individuals' SARS-CoV-2-related beliefs, attitudes, and practices, particularly how these vary across sub-populations, is critical to the refinement and promulgation of public health measures designed to help mitigate viral transmission, protect those who are most vulnerable to severe disease or death, prevent overextension of hospital resources, and reduce economic and mental health hardships brought on by the pandemic. Though many studies have measured SARS-CoV-2-related knowledge, attitudes, and practices, these studies have occurred almost exclusively outside of the US (4–12). Generalization of these studies to a US population is not reasonable, given governmental and cultural differences between nations.

Aslan et al. (13) conducted a US national survey in early April 2020 that measured COVID-19 related incidence, knowledge, and behaviors. This study showed significant knowledge, practice, and infection outcome differences along sex, age, and race strata, with men and African Americans demonstrating less COVID-19-related knowledge and higher likelihood of infection than their female or White counterparts. In March 2020, Clements (14) surveyed Americans, showing that responses to the nascent crisis were divided along age and political lines, and that females were more knowledgeable about COVID-19 than males. Bailey et al. (15) conducted a survey among high-risk US adults in two-phases in mid- and late-March 2020, but did not observe significant gaps in multivariable models for perceived susceptibility, knowledge, reported behaviors, or preparedness between females and males. In June 2020, Czeisler et al. (16) reported that females were more likely than males to agree that indoor dining should be prohibited and were less likely to have been to a public place the preceding week. Wolf et al. (17) reported that females with chronic conditions expressed a higher seriousness of threat from SARS-CoV-2 than their male counterparts, a finding that persisted in multivariable analyses. Galasso et al. (18) conducted a study across eight countries, including the US, in which female respondents were more likely than male respondents to perceive COVID-19 as a serious health threat and to adhere to the pandemic-related restrictions enacted in their country. To our knowledge, there is otherwise limited US data on SARS-CoV-2 pandemic related beliefs, attitudes, and practices.

The primary goal of this study was to explore the relationship between self-reported binary gender identity (female/male) and beliefs, attitudes, and practices related to SARS-CoV-2. Our study aimed to investigate threat perception in the early stages of the pandemic to help fine tune public health mitigation efforts in the future. Using survey data from patients of our community healthcare system who were tested for SARS-CoV-2 in the first 3 months of the pandemic, we analyzed gendered responses to SARS-CoV-2-related threat perception, a sense of personal agency in mitigating viral spread, and attitudes toward four key practices of social distancing, remaining outdoors, handwashing, and masking. We also compared other pandemic-related behaviors including news consumption, having contact with known or suspicious cases, and pandemic-related key practices in remaining outdoors, handwashing, and masking between genders.

## Methods

### Data and Study Subjects

MultiCare Health System (MHS) is a not for profit, community-based healthcare system serving patients across Washington State. With eight hospitals and hundreds of primary, specialty, and urgent care clinics in both Eastern and Western Washington, our patient population is representative of the larger state population. Data for all patients tested for SARS-CoV-2 throughout MHS from initiation of testing (March 5, 2020) through termination of the state's initial stay-home order (19) (June 7, 2020) were extracted from our electronic health record (EHR) data repository. All adult patients whose EHR vital status at the time of survey distribution (July 2020) was alive or unknown, who had a documented email address, and whose EHR-documented language suggested English language use were considered eligible for inclusion. Using the REDCap Survey platform (20), patients were sent an email invite with an embedded link that opened to an e-consent form. Once complete, the e-consent redirected the respondent to the survey. Initial study invitation emails were sent on July 9, 2020, with reminder messages sent three times, at 5-day intervals. Data were frozen and the survey was closed 31 days after initial invitation. Data from completed surveys were merged with clinical data to capture the date of each person's SARS-CoV-2 test to allow for the consideration of time in the rapidly evolving context of the early pandemic response in Washington State.

The survey comprised four domains, including: (1) symptoms and test information; (2) SARS-CoV-2 related beliefs, attitudes, practices (social distancing, remaining outdoors, hand washing, and masking); (3) other pandemic-relevant behaviors, such as frequency of news consumption or leaving the house to obtain food or medical care; and (4) demographic information. Respondents were asked 36–62 questions, dependent on branching and skip choices, and were expected to complete the survey in 15 min. Understanding that recall bias would be unavoidable in the context of surveying patients who had been tested 4–16 weeks prior to survey administration, questions were carefully worded to repeatedly emphasize that respondents were being asked to answer the questions with respect to the 2 weeks prior to their SARS-CoV-2 test. Respondents who were tested more than once during the study period were asked to consider their first positive test, if applicable, or their first test, if all tests were negative. No incentive was offered for participation. The study was approved by the healthcare system's Institutional Review Board.

SARS-CoV-2 positivity was reported by the respondent and cross-referenced with the result for that patient in the EHR. During the initial months of the pandemic that are represented by the study period, testing for SARS-CoV-2 was typically restricted to symptomatic individuals with an exposure risk. Toward the end of the study period, our system began testing asymptomatic pre-operative and obstetric patients or those with a known or suspected SARS-CoV-2 exposure. As such, for both the survey and EHR testing data, inconclusive results were coded as positive, as any person with symptoms and an inconclusive test would have been managed as positive in the clinical setting.

### Survey Development and Structure

The survey was developed based on resources available early in the pandemic, including a suite of COVID-19 relevant survey tools compiled by the NIH Office of Behavioral and Social Sciences Research (21) and the PhenX Toolkit developed by the Understanding America Study (22). Six SARS-CoV-2-related statements were included in the survey and respondents were asked to choose from a 5-item Likert-type scale (strongly disagree, disagree, neither agree nor disagree, agree, strongly agree) to express level of agreement with each statement. Binary variables were derived to indicate agreement (agree or strongly agree) or disagreement (neutral, disagree, strongly disagree) with each statement. For the purpose of sensitivity analyses, an additional variable was created for which only *strongly agree* responses were considered positive for agreement.

The six focal statements comprised three beliefs (perceived level of threat to self, perceived threat to family, and perceived level of the impact of personal practice on viral transmission) and three attitudes (need for social distancing, handwashing, and masking to reduce viral transmission). Practices were assessed for all respondents but were only compared among those who had visited with people outside of their social distancing circle (those with whom they had contact of <6 feet for more than 10 min) in the 2 weeks prior to their test.

Additional information including the size of each respondent's social distancing circle, whether or not the respondent lived with others, frequency (>once per day, once per day, 2–5 times per week, once per week, not at all) of SARS-CoV-2-relevant news consumption, and self-reported healthcare worker status were also collected.

Demographic variables included gender, age (measured in years as a continuous variable), ethnicity, annual household income (derived as a binary variable of income ≥75k, the state's most recently measured median income), and binary variables indicating attainment of an undergraduate degree and presence of any comorbidity/health conditions (lung disease, heart disease, chronic kidney disease, diabetes, cancer treatment within the last 5 years, otherwise immunocompromised, or pregnant).

Race/ethnicity was gathered as a multi-category variable. Respondents were asked to indicate all ethnicities with which they identified. Due to numerous reports in the scientific and non-scientific literature documenting the differential impact of SARS-CoV-2 on communities of color (23–27), we derived a binary race/ethnicity variable that identified as People of Color all respondents who self-identified as one or more of the Black, American Indian or Alaska Native, Asia, Hispanic/Latino, Middle Eastern, Multi-racial, Native Hawaiian or Pacific Islander, or Other categories.

We use the term gender rather than sex because we asked respondents to report their gender identity, as opposed to their sex assigned at birth. In the survey, respondents were asked to indicate their gender identity, including Transgender, Non-binary, and Other gender identity; seven transgender and seven non-binary or other gender identity individuals responded. These respondents were not included in the present analysis due to its focus on binary gender identity but will be included in other analyses of these data.

### Statistical Analysis

**Figure 1:**
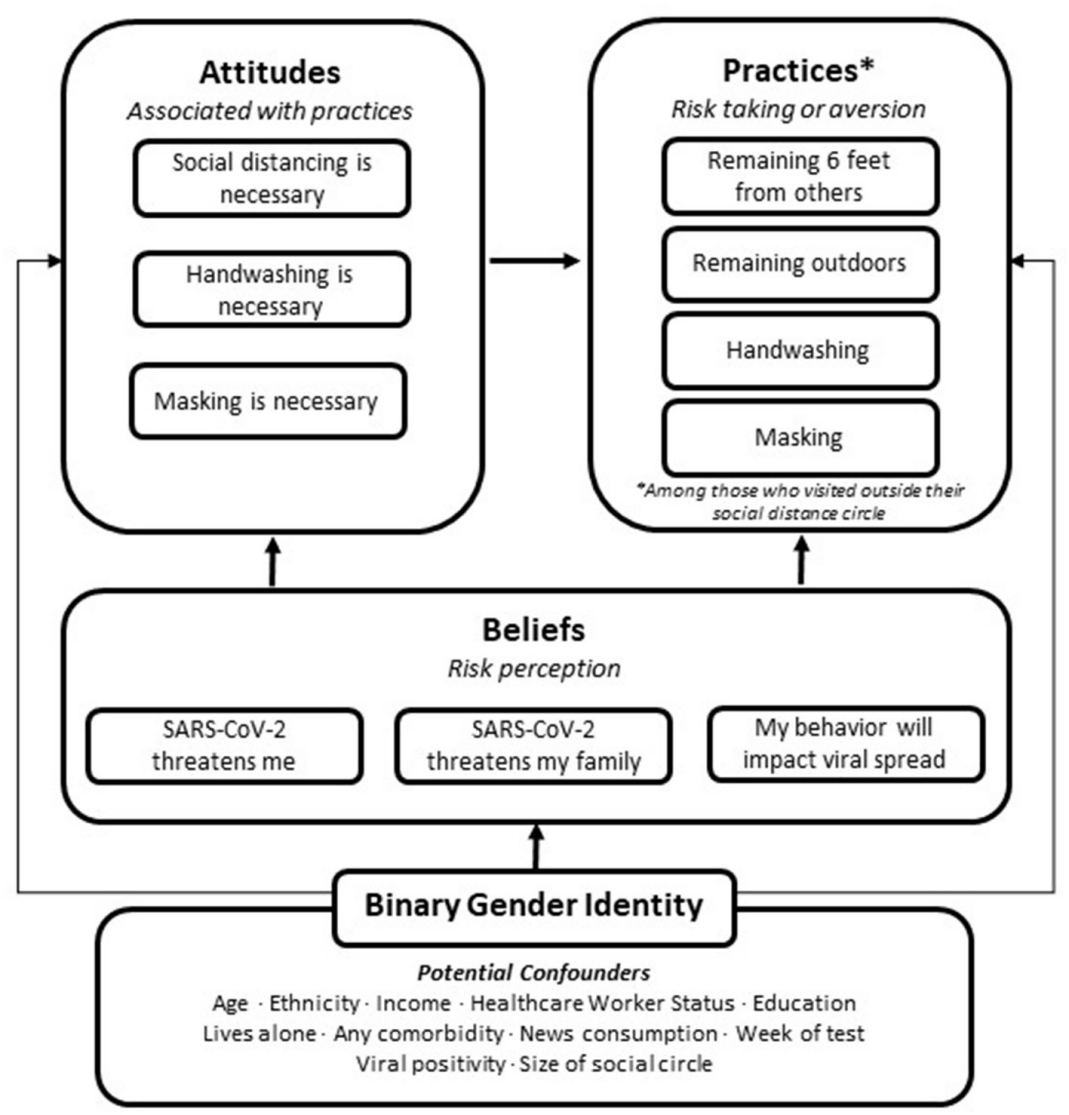
Conceptual framework for the relationship between binary gender identity and early-stage pandemic beliefs, attitudes, and practices.

The purpose of the present analysis was to understand respondents' SARS-CoV-2-related beliefs, attitudes, and practices in relation to binary gender identity (female/male), adjusting for potential confounding factors. We conceptualized beliefs as moderators of both attitudes and practices, and conceptualized attitudes as intermediary moderators of practices. In our conceptual framework (Figure 1), gender identity has moderating effects on each domain of interest and is intersectionally situated with potential confounders.

We examined the bivariate associations between gender and various demographics, presenting percent positive per variable category or mean with standard deviation and using Chi-squared tests of association and Student's *t*-tests, as appropriate. We used multivariable logistic regression to evaluate the relationship between gender and the outcomes of agreement with each of the six belief and attitude statements or pandemic-related practices, controlling for relevant sociodemographic factors or other important covariates that may correlate to gender and belief/attitudes/practices. To account for the potential bias introduced by one's test result, we further controlled all models for viral positivity.

To build multivariable logistic regression models, we began with a fixed set of potential confounding variables that were selected based on the literature and epidemiologic context. Using a manual, iterative process that combined scientific expertise and review of relevant model diagnostics, we then calibrated each model individually to determine the best ensembles of variables for understanding each outcome. We reviewed model diagnostics, including binned residual plots and variance inflation factors, selected final models that minimized the Akaike Information criteria, and used Hosmer-Lemeshow tests to confirm the adequacy of the final fitted models (28). The final covariates included in each model is presented in tables with results comparing females and males (reference gender group). Full model results are available as Supplementary Tables. Sensitivity analyses were performed for each model using belief and attitude variables for which only *strongly agree* was considered agreement. To compensate for the burden of multiple testing, *p* < 0.01 were considered statistically significant. Model building, data analysis, and data visualization were performed in the R statistical computing environment (Vienna, Austria; version 4.1.0).

## Results

During the study period, SARS-CoV-2 tests were administered to 23,455 adults (≥18 years old) who were believed to be alive at the time of survey distribution (July 9, 2020). Of those, 22,674 (97.2%) were believed to be English language users based on EHR language documentation and 15,730 (69.4%) of those patients had an email address in the EHR. Survey invitations were undeliverable to 2,308 patients and were thus believed to have been successfully delivered to 13,422 individuals. In the 31 days that the survey was open, 1,571 individuals responded, for a final response rate of 11.7%. Compared to the population of tested adults, respondents represented significantly more Whites (81.8 vs. 67.3%), females (72.6 vs. 60.5%), and older people (mean age 51.3 ± 16.3 vs. 47.7 ± 18.1).

Self-reported and EHR SARS-CoV-2 test results were in agreement for 98.4% of the respondents (*n* = 1,546). Twenty-five subjects reported positive or inconclusive results but had a negative result documented in the EHR. Given the overwhelming agreement between self-report and EHR, along with wide-ranging estimated false negativity of SARS-CoV-2 tests (29), we used the patient self-report test result in this analysis. A lower percentage of females (8.3%) than males (12.3%) reported viral positivity, though this difference was not significant at the 0.01 level (*p* = 0.02). Thirty-one females (2.8%) and 21 males (5.0%; *p* = 0.05) in our sample were hospitalized due to COVID-19. Survey respondents were tested across the course of the study period, with a positivity distribution that peaked in weeks 3 through 5, mirroring the distribution of the tested population. The overall percentage of viral positivity (9.4%) among respondents was higher than that of the population of all tested adults (5.4%).

A significantly higher proportion (72.6%, *p* < 0.001) of respondents described themselves as female than male (Table 1). The only two baseline characteristics in this voluntary sample that differed by gender were mean age (female: 49.6 ± 15.8 years, male: 51.3 ± 16.4 years), and healthcare worker status (female: 32.2%, male: 12.8%). Female and male respondents were comparable in other characteristics, including: held undergraduate degrees (47.7% overall), identified as People of Color (18.4% overall), had any comorbidity (39.2% overall), were asymptomatically tested for SARS-CoV-2 (34.4%), had a social-distance circle that included people outside their home (39.2% overall), and lived alone (13% overall).

**Table 1:** Respondent characteristics by binary gender identity.

	**Female**	**Male**	**Total**	
**Characteristic**	***n*** **(%)**			***p***
Respondents	1,122 (72.6)	423 (27.4)	1,545 (100)	<0.001
People of color	211 (19.0)	70 (16.8)	281 (18.4)	0.35
Any comorbidity	649 (57.8)	234 (55.3)	883 (57.9)	0.40
Healthcare worker	381 (32.2)	54 (12.8)	435 (27.1)	<0.001
Undergraduate degree	519 (46.8)	211 (50.1)	730 (47.7)	0.27
Income over WA state median	582 (53.8)	248 (61.4)	830 (55.9)	0.01
SD circle includes people outside the home	455 (40.6)	150 (35.5)	605 (39.2)	0.08
Lives alone	150 (13.4)	50 (11.8)	200 (13.0)	0.46
Tested positive for SARS-CoV-2	93 (8.3)	52 (12.3)	145 (9.4)	0.02
	**Female**	**Male**	**All**	
	**Mean (SD)**			***p***
Number of people outside the home included in SD circle	2.27 (3.4)	2.27 (3.6)	2.26 (3.4)	0.99
Total number of people in SD circle	4.32 (3.6)	4.05 (3.6)	4.25 (3.6)	0.19
Mean age	49.6 (15.8)	56.3 (16.4)	51.3 (16.3)	<0.001

Behaviors reported by female and male respondents in the 2-weeks prior to their SARS-CoV-2 tests are shown in Table 2. More females (18.5%) than males (10.9%, *p* < 0.001) reported having contact with a known case or a person with COVID-like symptoms, though this difference did lessen when healthcare workers were excluded (10.4 vs. 7.3%, *p* = 0.11). There were significant differences in reported access of news about the virus leading up to SARS-CoV-2 testing. Fewer female (49.6%) than male (54.6%) respondents accessed news more than once a day, and more female respondents accessed news only once per week (6.2 vs. 5.2%, *p* = 0.02). More females accessed news *via* social media (55.2%) compared to males (43.7%, *p* < 0.001).

**Table 2:** Respondent behaviors in the 2-weeks prior to SARS-CoV-2 viral test.

**Behaviors**	Female *n* (%^*^)	Male*n* (%^*^)	Total *n* (%^*^)	***p***
Possible or known exposure	208 (18.5)	46 (10.9)	254 (16.4)	<0.001
As above, excluding healthcare	84 (10.4)	27 (7.3)	111 (9.5)	0.11
workers				
Entered another person's home	267 (23.8)	103 (24.3)	370 (23.9)	0.87
Had others in home	269 (24.6)	106 (25.1)	375 (24.7)	0.64
Gathered with >10 people	119 (10.6)	49 (11.6)	168 (10.9)	0.65
News consumption frequency				
More than once per day	536 (49.6)	226 (54.6)	762 (51.0)	0.02
Once per day	349 (32.3)	135 (32.6)	484 (32.4)	
2–5 times per week	129 (11.9)	42 (10.1)	171 (11.4)	
Once per week	67 (6.2)	11 (2.7)	78 (5.2)	
Did not access news	37 (3.3)	9 (2.1)	46 (3.0)	0.14
Any national/local news access vs.	1,010 (90.0)	390 (92.2)	1,400 (90.6)	0.23
none				
Accessed news ≥ 1x per day	885 (81.9)	361 (87.2)	1,246 (83.3)	0.02
	**Female**	**Male**	**All**	
	**Mean (SD)**			***p***
Number of days left home to work	4.23 (4.7)	4.07 (4.9)	4.19 (4.7)	0.56
Number of days left home to get food	3.35 (2.9)	3.76 (3.1)	3.46 (3.0)	0.02
Number of days left home to socialize	1.42 (2.3)	1.6 (2.7)	1.48 (2.4)	0.24

* *Percentages provided per gender category*.

We observed significant differences between female and male responses to all the belief statements and two of the three attitude statements. In every instance, female respondents were more likely to agree with the statement. Adjusted odds of females agreeing that SARS-CoV-2 posed a personal threat was 1.51 times that of males (95% CI 1.14–2.00, *p* = 0.004) and females had 1.75-fold higher odds (95% CI 1.31–2.33, *p* < 0.001) of agreeing that their family was significantly threatened by SARS-CoV-2. Finally, odds of females agreeing that their behaviors will influence viral spread was more than double that of males (aOR = 2.17, 95% CI 1.49–3.15, *p* < 0.001). See Table 3 for the full text of belief statements and covariates retained in the final models.

**Table 3:** Adjusted odds ratios, 95% confidence intervals, and covariates for multivariable logistic regression models assessing the relationship between gender identity and SARS-CoV-2 (a) beliefs, (b) attitudes, and (c) practices.

**Outcome**	**aOR (95% CI)^¶^**	***p***	Covariates (^*^covariate with *p* < 0.01)
**(a) Beliefs**			
*Coronavirus poses a significant threat to me*.	1.51 (1.14–2.00)	0.004	Age^*^, People of color, Income over state median, Any comorbidity^*^, Healthcare worker, Week of test^*^, Frequency of news consumption^*^, Size of social distancing circle, Viral positivity
*Coronavirus poses a significant threat to my family members*.	1.75 (1.31–2.33)	<0.001	Age^*^, People of Color, Income over state median, Undergraduate degree, Any comorbidity^*^, Employed, Viral positivity^*^, Frequency of news consumption^*^, Size of social distancing circle^*^, Lives with others
*How I behave will make a difference in the spread of coronavirus*.	2.17 (1.49–3.15)	<0.001	Age, People of color, Income over state median, Undergraduate degree, Healthcare worker, Week of test, Frequency of news consumption^*^, Size of social distancing circle^*^, Lives with others, Viral positivity
**(b) Attitudes**			
*Social distancing is necessary to slow the spread of coronavirus*.	1.72 (1.19–2.46)	0.004	Age^*^, People of color, Income over state median, Undergraduate degree, Frequency of news consumption^*^, Size of social distancing circle^*^, Viral positivity
*Frequent hand washing is necessary to slow the spread of coronavirus*.	3.27 (2.06–5.21)	<0.001	Age, People of color, Income over state median, Undergraduate degree, Viral positivity, Healthcare worker, Week of test, Frequency of news consumption, Size of social distancing circle, Lives with others
*Wearing a mask helps slow the spread of coronavirus*.	1.41 (1.02–1.94)	0.034	Age, People of color^*^, Income over state median, Undergraduate degree, Any comorbidity, Frequency of news consumption^*^, Size of social distancing circle^*^, Lives with others, Viral positivity
**(c) Practices**			
*In the 2 weeks before you were tested for coronavirus, did you visit with people who live outside of your home or social distance circle?*	0.62 (0.47–0.81)	<0.001	Age, People of color, Income over state median, Undergraduate degree, Employed, Healthcare worker, Week of test^*^, Frequency of news consumption, Size of social distancing circle^*^, Viral positivity
^∞^ *Did you maintain six feet of distance at all times?*	1.41 (0.89–2.23)	0.14	Age^*^, People of color, Income over state median, Any comorbidity, Viral positivity, Week of test^*^, Frequency of news consumption, Size of social distancing circle^*^
^∞^ *Did you remain in an outdoor area?*	0.89 (0.56–1.40)	0.60	Age, People of color, Income over state median, Undergraduate degree, Employed, Week of test^*^, Frequency of news consumption, Size of social distance circle^*^, Viral positivity
^∞^ *Did you wear a mask?*	1.19 (0.74–1.93)	0.48	Age, people of color, Income over state median, Undergraduate degree, Any comorbidity, Week of test^*^, Frequency of news consumption, Size of social distance circle^*^, Viral positivity
^∞^ *Did you wash your hands with soap for 20 s afterwards?*	2.11 (1.33–3.34)	0.001	Age, People of color, Income over state median, Undergraduate degree, Week of test^*^, Frequency of news consumption, Size of social distance circle^*^, Viral positivity

*Outcomes are shown as worded in the survey*.

¶ *Reference group: men*.

∞ *Among those who visited outside their social distance circle*.

* *Covariate with significant association in the final model*.

Female respondents were also more likely to agree that public health mitigation measures are necessary to slow the spread of SARS-CoV-2. Odds of females agreeing that social distancing is necessary was 1.72 that of males (95% CI 1.19–2.46, *p* = 0.004). In the model related to the need for handwashing to slow the spread of SARS-CoV-2, which adjusted for healthcare worker status, odds of female agreement were more than 3-fold higher than male agreement (aOR = 3.27, 95% CI 2.06–5.21, *p* < 0.001); notably, no other covariate was significantly associated with odds of agreement with this statement. The need for masking was the lone exception in significant differences at the 0.01 level between females and males, but even in this non-significant finding, odds remained elevated for females (aOR = 1.41, 95% CI 1.02–1.94, *p* = 0.034).

Females had significantly lower odds than males of visiting with individuals outside of their social distance circle (aOR = 0.62, 95% CI0.47–0.81, *p* < 0.001). Among the one-third (*n* = 527) of respondents who did engage in visits outside of their social distancing circles, differences in pandemic-related practices were generally not significantly different. Odds of maintaining a distance of 6-feet or more was comparable (aOR = 1.41, 95% CI 0.89–2.23, *p* = 0.14), as were odds of remaining outdoors (aOR = 0.89, 95% CI 0.56–1.40, *p* = 0.60) and masking (aOR = 1.20, 95% CI 0.75–1.95, *p* = 0.43). Only the practice of handwashing with soap for 20 s following visits differed between females and males who visited outside of their social distancing circle, with females twice as likely to have done so compared to males (aOR = 2.11, 95% CI 1.33–3.34, *p* = 0.001).

Estimated odds ratios in sensitivity analyses (see Supplementary Table 2), for which agreement was only considered to be those who strongly agreed with each given statement, were stable for every belief and attitude. Odds of females strongly agreeing that handwashing is necessary decreased slightly in the sensitivity analysis (aOR = 2.23, 95% CI 1.71–2.91, *p* < 0.001), compared to the primary analysis (aOR = 3.27). The odds of females strongly agreeing with the need for masking was consistent with the primary analysis (aOR = 1.41, 95% CI 1.09–1.85) and was statistically significant (*p* = 0.009) in the sensitivity analysis.

## Discussion

This study is among the first in the United States to examine binary gender identity based discrepancies in SARS-CoV-2 related beliefs, attitudes, and practices. This survey was completed in Washington State by respondents who were tested for the virus across the initial months of the pandemic. During these months, national public health messaging was contradictory and inconsistent, particularly with respect to masking. These initial pandemic months also represented a knowledge-deficient time during which new information about and understanding of SARS-CoV-2 were literally updated daily. Simultaneously, disinformation, including conspiracy theories related to SARS-CoV-2, spread rapidly through social media and select media outlets and, in some instances, were propagated by prominent public figures and members of local and national government. This brought a substantial level of political divisiveness to the pandemic response and created barriers for public health mitigation efforts. These factors represent the broader social context of the months upon which this study focused.

Despite available information that male sex was a risk factor for hospitalization and death (30, 31), male respondents in our sample had higher viral testing positivity, significantly lower odds of perceiving threat, personal agency, and attitudes of agreement toward public health recommendations. Males were more likely to engage in visits with people outside their immediate social distancing circle, in contrast to public health recommendations, and were less likely to handwash following those visits, but did not differ significantly from female respondents in social distancing, remaining outdoors, or masking during the visits. This suggests a disconnect between public health messaging and pandemic-related beliefs, attitudes, and key practices among males. These findings are consistent with other studies and polls from early in the pandemic demonstrating that men are less likely than women to engage in hand hygiene practices (32–34), are less concerned than women that they or their family members will be exposed to SARS-CoV-2 (35), and are more likely to downplay the severity of the virus (36). Griffith et al. (37) discuss the intersection of biologic and health-practice related risk factors for men and the roots of these psychosocial risk factors in the social construct of masculinity, highlighting the importance of public health tactics that engage a more wholistic approach to men's health in addressing male responses to the SARS-CoV-2 pandemic.

Altogether, this information suggests that current public health messaging surrounding SARS-CoV-2 may be insufficient in effectively engaging large swaths of the population. Females in this study were significantly more likely to perceive threat or a sense of agency and agree that various public health mitigation strategies are important. This may reflect our national trend of overburdening of women in familial and emotional labor, which has been exacerbated by the pandemic, and calls attention to the need to explicitly target public health messaging toward men, both in content and media/messaging channels. Information from this study also highlights the particularly gender-discrepant attitudes and practices surrounding handwashing, which both supports prior findings and elevates the urgency for targeted efforts in this arena.

## Limitations

The primary limitations of this study are the multiple sources of potential bias. The sample is voluntary and thus does not account for the higher likelihood that female and test-positive subjects will respond to surveys and participate in research than men and test-negative subjects, nor does a voluntary survey adequately sample from a population. The study is also limited in its reliance on retrospective recall, a potential source of bias. Recall bias can be difficult to avoid, particularly in the context of a newly emerging infectious disease. Deeply seated in the pandemic now, future research will allow for better reduction of recall bias by closing the gap between SARS-CoV-2 testing and survey administration. The distribution method of electronic-only surveys in English-only likely results in the loss of important voices, particularly in light of the differential impact of SARS-CoV-2 in communities of color and immigrant communities (38). Finally, the study was conducted at the beginning of the pandemic, during a time in which masking was both not required and highly contentious with wildly conflicting messaging over the course of the brief course of the study period. With research and development rapidly updating and nearly 50% of the population fully vaccinated at the time of this writing, the current social and medical contexts differ from those of the study period. While these issues certainly reduce generalizability, it remains beneficial to examine current pandemic issues through the lens provided by this study, which suggests differential orientations amongst women and men in the early stages of this infectious disease crisis.

Further research into the knowledge, attitudes, and practices surrounding SARS-CoV-2 is extremely important, especially in the current climate of plateauing vaccination rates. Such work should be prospective, purposefully sampled, ideally using mixed-methods approach. Results from prospective and purposefully sampled studies will be of great value in informing public health measures both with respect to gender identity and in efforts to address the gross health inequities that exist in this pandemic and otherwise.

## Data Availability Statement

The raw data supporting the conclusions of this article will be made available by the authors, without undue reservation.

## Ethics Statement

The studies involving human participants were reviewed and approved by MultiCare Health System Institutional Review Board. The patients/participants provided their written informed consent to participate in this study.

## Author Contributions

Both authors contributed to the study design, data collection, analysis, and preparation and approval of the final manuscript.

## Funding

This study was funded with institutional resources.

## Conflict of Interest

The authors declare that the research was conducted in the absence of any commercial or financial relationships that could be construed as a potential conflict of interest.

## Publisher's Note

All claims expressed in this article are solely those of the authors and do not necessarily represent those of their affiliated organizations, or those of the publisher, the editors and the reviewers. Any product that may be evaluated in this article, or claim that may be made by its manufacturer, is not guaranteed or endorsed by the publisher.
