# Supplementary material for: Gender Discrepancies in SARS-CoV-2 Pandemic Related Beliefs, Attitudes, and Practices
Source: Front Public Health. 2021 Sep 27;9:711460. doi: 10.3389/fpubh.2021.711460 (PMC8502889; doi:10.3389/fpubh.2021.711460)
Supplement: Supplementary file 1 [file Table_1.DOCX]

Supplementary Material

**S1(a-f).** Full results of multivariable models with outcomes of agreement with coronavirus-related beliefs (a-c), attitudes (d-f), and practices (g-k).

| **a)** Coronavirus poses a significant threat to me. | **aOR** | **LCI** | **UCI** | **p** |
| --- | --- | --- | --- | --- |
| Females | 1.51 | 1.14 | 2.00 | 0.004 |
| Age | 1.03 | 1.02 | 1.03 | 0.000 |
| People of Color | 1.33 | 0.96 | 1.85 | 0.090 |
| Income over state median | 0.84 | 0.65 | 1.08 | 0.166 |
| Any comorbidity | 2.45 | 1.91 | 3.15 | 0.000 |
| Healthcare worker | 1.28 | 0.95 | 1.74 | 0.108 |
| Week of test | 0.96 | 0.93 | 0.99 | 0.009 |
| News consumption: Once per day | 0.59 | 0.45 | 0.78 | 0.000 |
| News consumption: 2-5 times per week | 0.55 | 0.38 | 0.82 | 0.003 |
| News consumption: Once per week | 0.27 | 0.16 | 0.46 | 0.000 |
| Size of social distancing circle* | 0.93 | 0.90 | 0.97 | 0.000 |
| Viral positivity | 1.10 | 0.73 | 1.68 | 0.660 |
|  |  |  |  |  |
| **b)** Coronavirus poses a significant threat to my family members. | **aOR** | **LCI** | **UCI** | **p** |
| Females | 1.75 | 1.31 | 2.33 | 0.000 |
| Age | 1.00 | 0.99 | 1.01 | 0.517 |
| People of Color | 1.18 | 0.83 | 1.69 | 0.368 |
| Income over state median | 0.84 | 0.63 | 1.12 | 0.231 |
| Undergraduate degree | 1.36 | 1.03 | 1.79 | 0.028 |
| Any comorbidity | 1.42 | 1.08 | 1.86 | 0.012 |
| Employed | 1.17 | 0.86 | 1.60 | 0.317 |
| Viral positivity | 1.98 | 1.22 | 3.35 | 0.008 |
| News consumption: Once per day | 0.75 | 0.55 | 1.01 | 0.059 |
| News consumption: 2-5 times per week | 0.66 | 0.44 | 1.02 | 0.054 |
| News consumption: Once per week | 0.24 | 0.14 | 0.40 | 0.000 |
| Size of social distancing circle* | 0.94 | 0.90 | 0.97 | 0.000 |
| Lives with others | 1.36 | 0.90 | 2.01 | 0.132 |
|  |  |  |  |  |
| **c)** How I behave will make a difference in the spread of coronavirus. | **aOR** | **LCI** | **UCI** | **p** |
| Females | 2.17 | 1.49 | 3.15 | 0.000 |
| Age | 1.00 | 0.99 | 1.01 | 0.685 |
| People of Color | 1.33 | 0.84 | 2.20 | 0.245 |
| Income over state median | 1.20 | 0.83 | 1.72 | 0.339 |
| Undergraduate degree | 1.32 | 0.92 | 1.92 | 0.134 |
| Healthcare worker | 1.31 | 0.83 | 2.12 | 0.260 |
| Week of test | 0.97 | 0.93 | 1.02 | 0.249 |
| News consumption: Once per day | 0.92 | 0.61 | 1.40 | 0.706 |
| News consumption: 2-5 times per week | 0.49 | 0.30 | 0.83 | 0.007 |
| News consumption: Once per week | 0.29 | 0.16 | 0.53 | 0.000 |
| Size of social distancing circle* | 0.90 | 0.86 | 0.95 | 0.000 |
| Lives with others | 0.62 | 0.32 | 1.13 | 0.138 |
| Viral positivity | 1.53 | 0.82 | 3.09 | 0.205 |
|  |  |  |  |  |
| **d)** Social distancing is necessary to slow the spread of coronavirus. | **aOR** | **LCI** | **UCI** | **p** |
| Females | 1.72 | 1.19 | 2.46 | 0.004 |
| Age | 1.00 | 0.99 | 1.01 | 0.913 |
| People of Color | 1.69 | 1.06 | 2.80 | 0.034 |
| Income over state median | 1.16 | 0.81 | 1.65 | 0.416 |
| Undergraduate degree | 1.35 | 0.95 | 1.94 | 0.094 |
| News consumption: Once per day | 0.88 | 0.60 | 1.31 | 0.532 |
| News consumption: 2-5 times per week | 0.55 | 0.33 | 0.92 | 0.019 |
| News consumption: Once per week | 0.38 | 0.21 | 0.71 | 0.002 |
| Size of social distancing circle* | 0.87 | 0.84 | 0.91 | 0.000 |
| Viral positivity | 0.70 | 0.37 | 1.22 | 0.227 |
|  |  |  |  |  |
| **e)** Frequent hand washing is necessary to slow the spread of coronavirus. | **aOR** | **LCI** | **UCI** | **p** |
| Females | 3.27 | 2.06 | 5.21 | 0.000 |
| Age | 1.00 | 0.99 | 1.01 | 0.984 |
| People of Color | 1.27 | 0.68 | 2.54 | 0.478 |
| Income over state median | 1.10 | 0.68 | 1.75 | 0.702 |
| Undergraduate degree | 1.41 | 0.88 | 2.27 | 0.151 |
| Viral positivity | 1.54 | 0.71 | 3.87 | 0.312 |
| Healthcare worker | 2.04 | 1.05 | 4.38 | 0.048 |
| Week of test | 1.02 | 0.96 | 1.08 | 0.565 |
| News consumption: Once per day | 1.07 | 0.65 | 1.80 | 0.780 |
| News consumption: 2-5 times per week | 1.43 | 0.66 | 3.60 | 0.402 |
| News consumption: Once per week | 0.48 | 0.22 | 1.18 | 0.087 |
| Size of social distancing circle* | 0.97 | 0.91 | 1.03 | 0.303 |
| Lives with others | 0.48 | 0.18 | 1.08 | 0.104 |
|  |  |  |  |  |
| **f)** Wearing a mask helps slow the spread of coronavirus. | **aOR** | **LCI** | **UCI** | **p** |
| Females | 1.41 | 1.02 | 1.94 | 0.034 |
| Age | 1.01 | 1.00 | 1.02 | 0.184 |
| People of Color | 2.17 | 1.45 | 3.36 | 0.000 |
| Income over state median | 1.17 | 0.86 | 1.58 | 0.312 |
| Undergraduate degree | 1.45 | 1.08 | 1.97 | 0.015 |
| Any comorbidity | 1.20 | 0.90 | 1.61 | 0.217 |
| News consumption: Once per day | 0.80 | 0.57 | 1.12 | 0.186 |
| News consumption: 2-5 times per week | 0.37 | 0.25 | 0.57 | 0.000 |
| News consumption: Once per week | 0.27 | 0.16 | 0.46 | 0.000 |
| Size of social distancing circle* | 0.90 | 0.87 | 0.93 | 0.000 |
| Lives with others | 0.61 | 0.36 | 1.00 | 0.060 |
| **g)** In the two weeks before you were tested for coronavirus, did you visit with people who live outside of your home or social distance circle? | **aOR** | **LCI** | **UCI** | **p** |
| Females | 0.62 | 0.47 | 0.81 | 0.000 |
| Age | 1.01 | 1.00 | 1.01 | 0.178 |
| People of Color | 0.66 | 0.47 | 0.91 | 0.012 |
| Income over state median | 0.93 | 0.72 | 1.20 | 0.570 |
| Undergraduate degree | 1.15 | 0.90 | 1.48 | 0.274 |
| Employed | 1.49 | 1.09 | 2.03 | 0.012 |
| Healthcare worker | 0.77 | 0.56 | 1.06 | 0.111 |
| Week of test | 0.94 | 0.91 | 0.97 | 0.000 |
| News consumption: Once per day | 1.28 | 0.98 | 1.67 | 0.075 |
| News consumption: 2-5 times per week | 1.13 | 0.75 | 1.67 | 0.561 |
| News consumption: Once per week | 1.36 | 0.79 | 2.32 | 0.261 |
| Size of social distancing circle* | 1.23 | 1.19 | 1.27 | 0.000 |
| Viral positivity | 1.03 | 0.69 | 1.53 | 0.873 |
|  |  |  |  |  |
| **h)** Did you maintain six feet of distance at all times? | **aOR** | **LCI** | **UCI** | **p** |
| Females | 1.41 | 0.89 | 2.23 | 0.142 |
| Age | 1.02 | 1.01 | 1.04 | 0.001 |
| People of Color | 0.91 | 0.50 | 1.64 | 0.751 |
| Income over state median | 0.67 | 0.44 | 1.03 | 0.066 |
| Any comorbidity | 1.72 | 1.12 | 2.65 | 0.013 |
| Viral positivity | 0.52 | 0.24 | 1.06 | 0.085 |
| Week of test | 1.15 | 1.09 | 1.22 | 0.000 |
| News consumption: Once per day | 1.03 | 0.65 | 1.63 | 0.895 |
| News consumption: 2-5 times per week | 0.56 | 0.27 | 1.12 | 0.106 |
| News consumption: Once per week | 0.57 | 0.22 | 1.39 | 0.230 |
| Size of social distancing circle* | 0.88 | 0.83 | 0.93 | 0.000 |
|  |  |  |  |  |
| **i)** Did you remain in an outdoor area? | **aOR** | **LCI** | **UCI** | **p** |
| Females | 0.89 | 0.56 | 1.40 | 0.601 |
| Age | 1.00 | 0.99 | 1.02 | 0.809 |
| People of Color | 1.10 | 0.59 | 2.00 | 0.762 |
| Income over state median | 0.96 | 0.62 | 1.51 | 0.873 |
| Undergraduate Degree | 1.58 | 1.02 | 2.47 | 0.041 |
| Employed | 0.65 | 0.40 | 1.06 | 0.081 |
| Week of test | 1.14 | 1.08 | 1.20 | 0.000 |
| News consumption: Once per day | 1.07 | 0.67 | 1.69 | 0.784 |
| News consumption: 2-5 times per week | 0.49 | 0.22 | 1.03 | 0.068 |
| News consumption: Once per week | 1.23 | 0.49 | 2.94 | 0.648 |
| Size of social distancing circle* | 0.90 | 0.85 | 0.95 | 0.000 |
| Viral positivity | 0.59 | 0.25 | 1.26 | 0.197 |
|  |  |  |  |  |
| **j)** Did you wear a mask? | **aOR** | **LCI** | **UCI** | **p** |
| Females | 1.19 | 0.74 | 1.93 | 0.477 |
| Age | 1.02 | 1.00 | 1.03 | 0.021 |
| People of Color | 1.09 | 0.56 | 2.04 | 0.802 |
| Income over state median | 0.90 | 0.57 | 1.43 | 0.655 |
| Undergraduate Degree | 0.56 | 0.35 | 0.89 | 0.015 |
| Any comorbidity | 1.91 | 1.21 | 3.05 | 0.006 |
| Week of test | 1.13 | 1.07 | 1.19 | 0.000 |
| News consumption: Once per day | 0.69 | 0.42 | 1.11 | 0.130 |
| News consumption: 2-5 times per week | 0.45 | 0.20 | 0.97 | 0.052 |
| News consumption: Once per week | 0.52 | 0.17 | 1.33 | 0.195 |
| Size of social distancing circle* | 0.89 | 0.84 | 0.95 | 0.000 |
| Viral positivity | 0.58 | 0.23 | 1.32 | 0.221 |
|  |  |  |  |  |
| **k)** Did you wash your hands with soap for 20 seconds afterwards? | **aOR** | **LCI** | **UCI** | **p** |
| Females | 2.11 | 1.33 | 3.34 | 0.001 |
| Age | 1.01 | 1.00 | 1.02 | 0.193 |
| People of Color | 2.26 | 1.24 | 4.34 | 0.011 |
| Income over state median | 0.92 | 0.59 | 1.42 | 0.704 |
| Undergraduate Degree | 0.58 | 0.37 | 0.89 | 0.014 |
| Week of test | 1.07 | 1.02 | 1.13 | 0.009 |
| News consumption: Once per day | 0.99 | 0.62 | 1.58 | 0.958 |
| News consumption: 2-5 times per week | 0.93 | 0.48 | 1.85 | 0.823 |
| News consumption: Once per week | 0.59 | 0.26 | 1.39 | 0.219 |
| Size of social distancing circle* | 0.91 | 0.86 | 0.95 | 0.000 |
| Viral positivity | 0.93 | 0.51 | 1.75 | 0.823 |

* Social distance circle: the people, outside of work, with whom the respondent comes closer than 6 feet for more than 15 minutes

| **S2**. Adjusted odds ratios, and covariates for multiple logistic regression models assessing the relationship between gender identity and (a) beliefs or (b) attitudes. Sensitivity analysis for which agreement with statement is restricted to *strongly agree* responses. | | | |
| --- | --- | --- | --- |
| **Outcome** | **aOR (95% CI) for Females*** | **p** | **Covariates** |
| 1. ***Beliefs*** |  |  |  |
| Coronavirus poses a significant threat to me. | 1.56 (1.18-2.06) | 0.002 | Age*, People of Color, Income over state median, Any comorbidity*, Healthcare worker, Week of test*, Frequency of news consumption*, Size of social distancing circle, Viral positivity |
| Coronavirus poses a significant threat to my family members. | 1.65 (1.28-2.13) | 0.000 | Age*, People of Color, Income over state median, Undergraduate degree, Any comorbidity*, Employed, Viral positivity*, Frequency of news consumption*, Size of social distancing circle*, Lives with others |
| How I behave will make a difference in the spread of coronavirus. | 2.01 (1.55-2.61) | 0.000 | Age, People of Color, Income over state median, Undergraduate degree, Healthcare worker, Week of test, Frequency of news consumption*, Size of social distancing circle*, Viral Positivity |
| ***(b) Attitudes*** |  |  |  |
| Social distancing is necessary to slow the spread of coronavirus. | 1.70 (1.31-2.20) | 0.000 | Age*, People of Color, Income over state median, Undergraduate degree, Frequency of news consumption*, Size of social distancing circle*, Viral positivity |
| Frequent hand washing is necessary to slow the spread of coronavirus. | 2.23 (1.71-2.91) | 0.000 | Age, People of Color, Income over state median, Undergraduate degree, Viral positivity, Healthcare worker, Week of test, Frequency of news consumption, Size of social distancing circle, Lives with others |
| Wearing a mask helps slow the spread of coronavirus. | 1.41 (1.09-1.85) | 0.009 | Age, People of Color*, Income over state median, Undergraduate degree, Any comorbidity, Frequency of news consumption*, Size of social distancing circle*, Lives with others, Viral positivity |
| * Reference group: men | | | |
